# Study of the Distribution of Radiative Defects and Reabsorption of the UV in ZnO Nanorods-Organic Hybrid White Light Emitting Diodes (LEDs)

**DOI:** 10.3390/ma4071260

**Published:** 2011-07-08

**Authors:** Ijaz Hussain, Nargis Bano, Sajjad Hussain, Yousuf Soomro, Omer Nur, Magnus Willander

**Affiliations:** Department of Science and Technology, Campus Norrköping, Linköping University, SE-60174 Norrköping, Sweden; E-Mails: ijaas@ifm.liu.se (I.H.); narba@itn.liu.se (N.B.); shussainawan@gmail.com (S.H.); muhso@itn.liu.se (Y.S.); omeno@itn.liu.se (O.N.)

**Keywords:** ZnO nanorods, cathodoluminescence, LEDs

## Abstract

In this study, the low temperature aqueous chemical growth (ACG) method was employed to synthesized ZnO nanorods to process-organic hybrid white light emitting diodes (LEDs) on glass substrate. Electroluminescence spectra of the hybrid white LEDs demonstrate the combination of emission bands arising from radiative recombination of the organic and ZnO nanorods (NRs). Depth resolved luminescence was used for probing the nature and spatial distribution of radiative defects, especially to study the re-absorption of ultraviolet (UV) in this hybrid white LEDs structure. At room temperature the cathodoluminescence (CL) spectra intensity of the deep band emission (DBE) is increased with the increase of the electron beam penetration depth due to the increase of defect concentration at the ZnO NRs/Polyfluorene (PFO) interface and probably due to internal absorption of the UV. A strong dependency between the intensity ratio of the UV to the DBE bands and the spatial distribution of the radiative defects in ZnO NRs has been found. The comparison of the CL spectra from the PFO and the ZnO NRs demonstrate that PFO has a very weak violet-blue emission band, which confirms that most of the white emission components originate from the ZnO NRs.

## 1. Introduction

Zinc oxide (ZnO) nanostructures possess a promising future owing to the variety of optical and electrical properties which are technologically useful for many nanoscale electronic and photonic devices. Fabrication of efficient devices based on ZnO nanostructures, such as light emitting diodes (LEDs), requires in-depth understanding of the luminescent properties and the distribution of the luminescent centers in ZnO nanostructures. However, the efforts to characterize ZnO nanostructures have produced large inconsistencies in the optical results, and up until now it appears that the luminescence properties vary with the structure morphology, growth methods and conditions [1].

ZnO possesses a large number of radiative intrinsic and extrinsic deep level defects [2,3]. Particularly ZnO, besides the ultraviolet (UV) emission, emits blue, green, yellow and red colors; which covers the whole visible region [3,4]. Therefore, the optical properties of ZnO have been extensively studied. ZnO typically exhibits one sharp UV peak and possibly one or two broad deep band emissions (DBE) due to deep radiative defects within the band gap [3,5]. The dominant emitted colors depend on the growth method and conditions; this implies that the emitted color lines can be controlled [3]. Hence ZnO has great potential to the development of intrinsic white light emitting diodes (LEDs). During the last few years the lack of a reliable and reproducible p-type doping in ZnO material with sufficiently high conductivity and carrier concentration has initiated an alternative approach to grow ZnO nanostructures on other inorganic/organic p-type substrates to realize ZnO-based p–n heterojunctions. In recent years ZnO nanorods-organic hybrid white LEDs has become one of the most exciting research areas. Hybrid materials promise good properties that may not easily be available from conventional materials such as combining the high flexibility of polymers with the structural, chemical and high functional stability of inorganic materials. This may lead to flexible electronic and photonic devices. Yin *et al*. reported that extended interface areas available in nano-hybrid thin films exhibit highly efficient charge transfer processes and strong optical effects that are not observed in homogeneous materials [6]. It has been reported that inorganic materials and organic materials could form a device to realize emissions from both kinds of materials [3]. Considering the emission, we are interested in the DBE from ZnO nanorods (NRs) as it can provide a spectrum that covers the whole visible range. Although a lot of results on the origin of the DBE have been published, no consensus was reached and the causes for the formation of these intrinsic/extrinsic defects and their spatial distribution are still controversial. The position and behavior of the DBE in ZnO NRs show strong dependency on the growth methods and growth conditions [2]. The free carrier concentration and luminescence efficiency are directly or indirectly related to deep level defects [3]. The performance of ZnO NRs based optoelectrical devices depend upon the defect chemistry, the distribution of defects along the ZnO NRs and especially the origin of specific emissions from ZnO NRs and the substrates. The optical properties of ZnO NRs have been measured using photoluminescence (PL) but the spatial resolution of the PL is limited due to the unwanted second and higher-order diffraction maxima and poor signal to noise ratio [7,8]. For detailed emission information and to explain the origin of the specific emission from specific small area, or even from individual nanostructures, a probe with high spatial and spectral resolutions is preferable. In this respect, depth resolved cathodoluminescence (CL) spectroscopy is a powerful technique for probing the nature of defects, both electronically and spatially on the nanoscale [7,9]. The information derived from this technique provides a tool to pilot the growth and the processing of ZnO NRs based opto-electronics. In addition the information on the physical origin and growth dependence of the electrically active defects can also be extracted [6].

In this article, electroluminescence (EL) and CL of ZnO nanorods-organic hybrid white LEDs grown on glass substrates by the low temperature aqueous chemical technique, are discussed collectively to verify the origin of specific radiative emissions. The EL spectrum reveals a broad emission band covering the whole visible range emerging from the radiative recombination from the polymer and from the ZnO nanorods. The CL microscopy was employed for detailed luminescence information, especially to investigate the origin of specific emissions, reabsorption of the UV and the distribution of radiative defects. The depth resolved CL observation suggests that some part of the UV emission might be reabsorbed by the ZnO and possibly converted into visible emission and consequently contributes to the enhancement of the visible emission. The information derived from the depth resolved luminescence provides a tool for the growth and processing the state-of-the-art hybrid white LEDs. The distribution of the radiative defects and reabsorption of the UV are responsible for the wide modification of the luminescence properties of hybrid white LEDs based on ZnO NRs. Therefore it is important to obtain a detailed information of the luminescence and verify the origin of specific emissions from ZnO nanorods-organic hybrid white LEDs.

## 2. Results and Discussion

**Figure 1:**
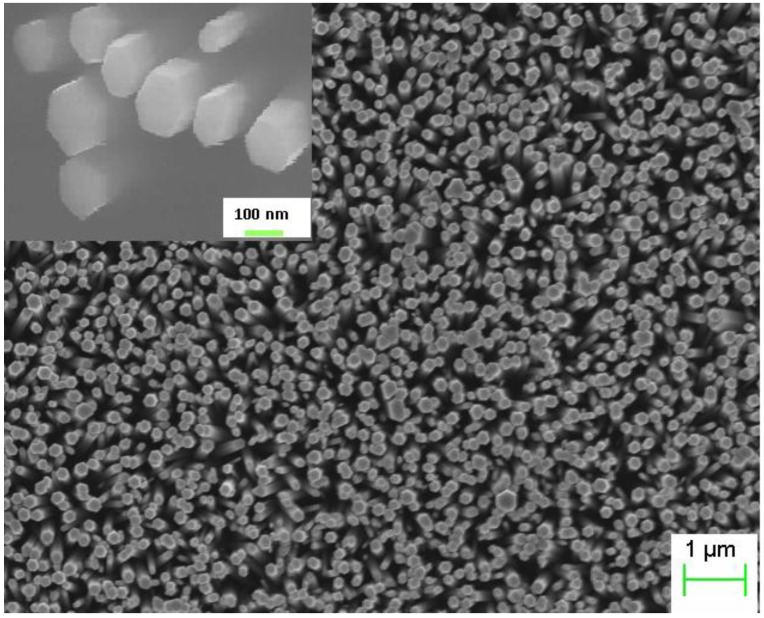
SEM image of the as grown ZnO nanorods (NRs) on Polyfluorene (PFO) polymer and the inset shows the SEM image after deposition of the spin on glass (SOG).

**Figure 2:**
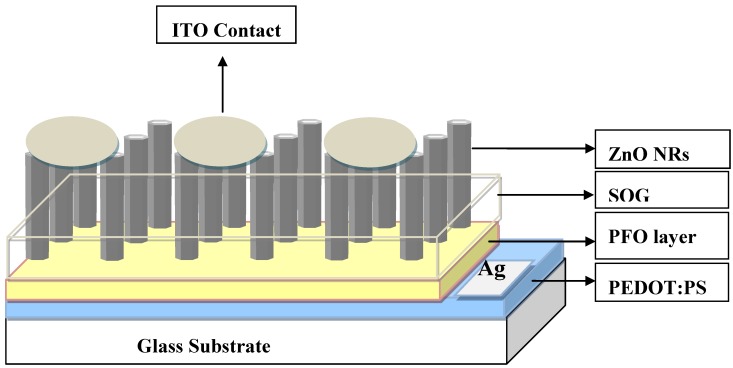
Schematic illustration of ZnO NRs/PFO/PEDOT: PSS/glass heterostructure device.

**Figure 3:**
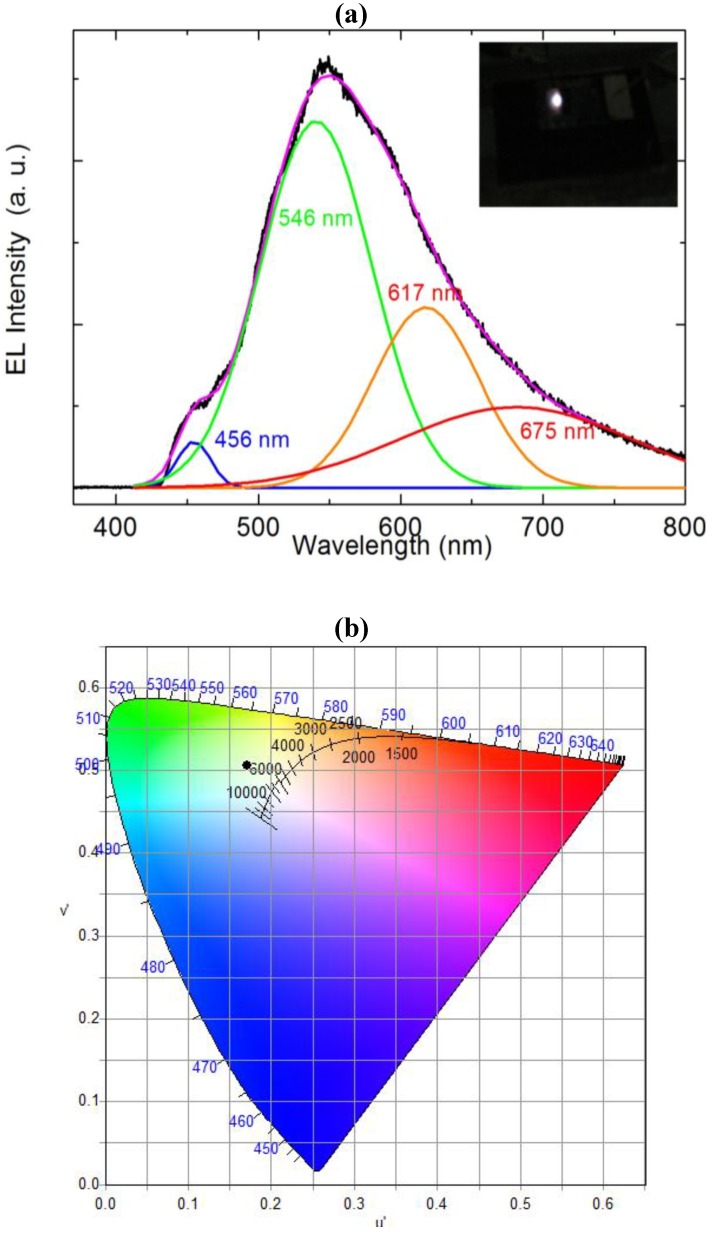
(**a**) Room temperature electroluminescence (EL) spectrum of the ZnO nanorods-organic hybrid white LEDs after Gaussian fitting and inset shows the photograph of white light emission from ZnO nanorods-organic hybrid white LED; (**b**) Chromaticity diagram of the ZnO nanorods-organic hybrid white LEDs.

The ZnO NRs grown on the top of the polymer were found to be vertically aligned and distributed uniformly with an average diameter and length 150 nm and 3.5 μm respectively as shown in Figure 1. The inset shows the SEM image after deposition of the spin on glass (SOG) insulating layer. A schematic diagram of the ZnO nanorods-organic hybrid white LED is shown in Figure 2. The RT-EL spectra of the ZnO nanorods-organic hybrid white LEDs reveal a broad emission band from 400 to 800 nm covering the whole visible region as shown in Figure 3. In order to distinguish the white-light EL spectra components we used a Gaussian function to simulate the experimental data. The simulated EL spectrum shows four emissions at 456 nm, 546 nm, 617 nm and 676 nm which are associated with blue, green, orange and red, respectively. It is clear that the emission at 456 nm (blue) is due to the radiative recombination from the Polyfluorene (PFO) and the green-red (546–675) emissions are associated with radiative defects in the ZnO. The EL photograph in the inset of Figure 3 shows the light emission is actually a white color impression. To investigate the color quality of the ZnO nanorods-organic hybrid white LEDs the color chromaticity coordinates are plotted in Figure 3(b). Figure 3(b) shows that the emitted light has a white impression. The color rendering index (CRI) and correlated color temperature (CCT) of the ZnO nanorods-organic hybrid white LEDs were calculated to be 81 and 5800 K, respectively.

**Figure 4:**
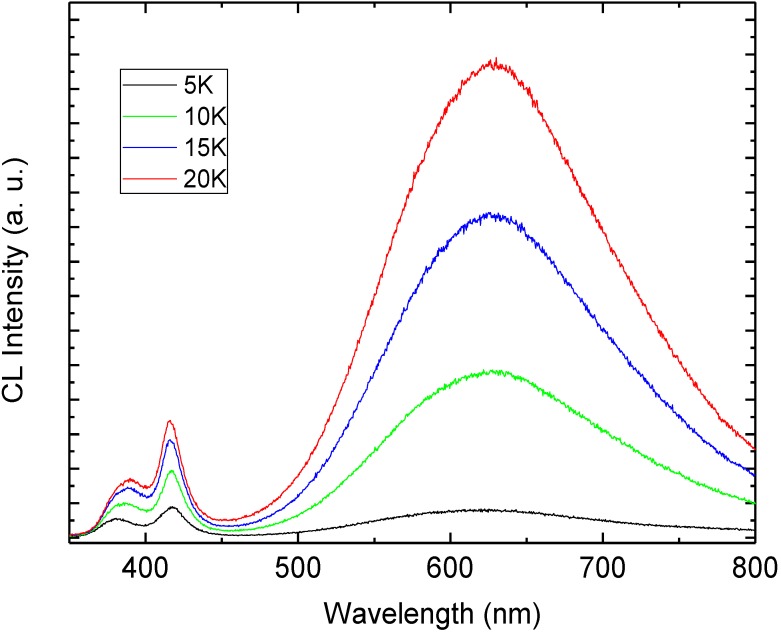
Depth dependent CL spectra of the hybrid LED at room temperature.

**Figure 5:**
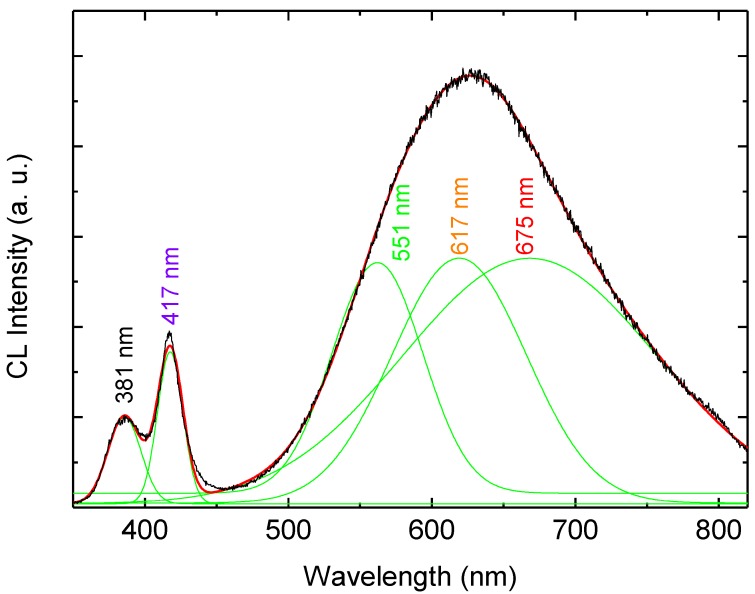
Room temperature CL spectrum of the hybrid LED after the Gaussian fitting.

To attain greater insight into the deep defect chemistry in the hybrid white LED structure, we measured the CL spectra at room temperature (RT). Figure 4 shows the RT-CL spectra at different accelerating voltages (5–20 kV). The number of excited carriers is considered to be in proportion to the accelerating voltage. The CL spectra exhibit UV emission at 381 nm which is related to the direct recombination of photon-generated charge carries (excitonic emission), and in addition to two DBE bands at 417 nm and 625 nm. In order to distinguish the emission components in the CL spectra we used a Gaussian function to simulate the experimental data as shown in Figure 5. The simulated CL spectrum shows five emissions at 381 nm, 417 nm, 551 nm, 617 nm and 676 nm which are associated with the UV, violet, green, orange and red, respectively. The violet emission is attributed to the transition from Zn_i_ to valance band [10]. The green luminescence band is the most investigated and most debated band in ZnO. Most recently, the green emission has been explained to asoriginating from more than one deep level defect, V_O_ and V_Zn_ with different optical characteristics were found to contribute to the broad green luminescence band [3,11,12]. Moreover, the orange emission was recently attributed to oxygen interstitial, while the red emission was proposed to be due to transition associated with zinc interstitials [3,13]. The Gaussian simulation gives clear evidence that the DBE band in the CL and the EL have the same emission lines but without the UV and the violet emissions in the EL spectra. Recently a blue emission from the PFO is reported at 446 nm but we observed at 456 nm this shift in the blue peak may be due to the contribution of violet emission form ZnO NRs [14]. However no consensus has been reached regarding the origin of the different observed colors, partly due to the different defect configuration in different samples grown by different methods [2,3,4]. Therefore, depth dependent CL spectra were examined to investigate the UV re-absorption and the detailed information of the luminescence and the spatial distribution of the defects that cause the UV and the wide visible emissions from the ZnO nanorods-organic hybrid white LEDs.

**Figure 6:**
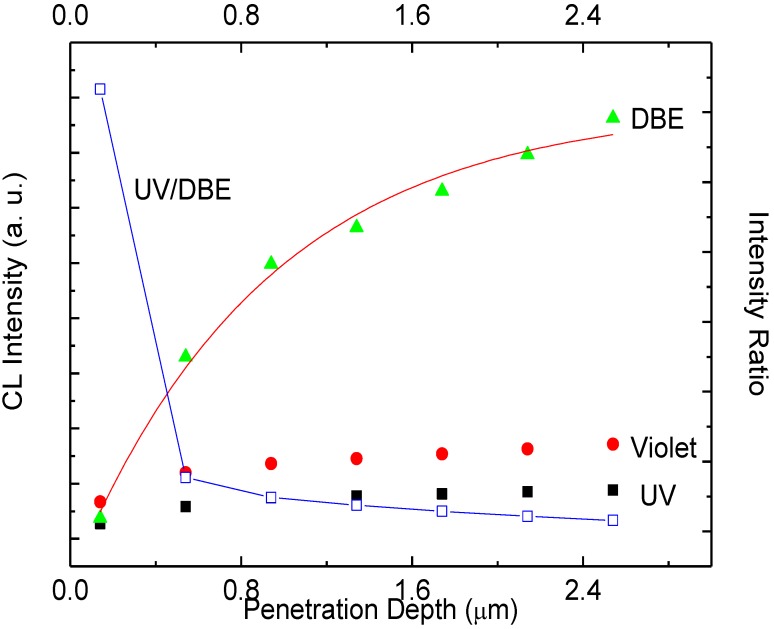
The emission intensity (right axis) of the UV, violet and DBE as well as the ratio of UV and DBE (left axis) as a function of penetration depth.

N. Bano *et al*. reported the energy loss per unit depth, dE/dx, as a function of depth into the target material (ZnO) for 20 kV and 30 kV accelerating voltages [15]. These curves present a polynomial fit to the Everhart-Hoff depth dose relation [16]. In order to realize the emission properties from ZnO NRs, it is necessary to clarify the effect of the electron beam conditions on the CL measurement. The penetration depth (R_e_) which is related to electron-hole pair creation rate, varies with the incident electron accelerating voltages such that R_e_ = 0.1, 0.4, 1.14 and 2.16 μm for V_b_ = 5, 10, 20 and 30 kV, respectively. An electron acceleration voltage of 20 kV corresponds to approximate 1μm of penetration depth [3,16]. The RT-CL spectra exhibit orders of magnitude lower UV and high DBE which shows that the defect concentration is varying in orders of magnitude with depth. The CL emission intensities of the UV and the DBE as a function of the penetration depth are shown in Figure 6. The UV and DBE show distinct differences in depth dependence. For a penetration depth between 0.54–2.14 μm the DBE intensity is higher than the UV intensity. The experimental DBE intensity data is compared with simulation (red line) the results obtained by exponential increase fitting procedure confirms that ZnO NRs have exponential distribution of defects along the nanorods as shown in Figure 6. By increasing the accelerating voltage, the electron beam is expected to penetrate deeper into the ZnO and excites more electron-hole pairs. In this way, more and more emission centers will be excited by the electron bombardment. It is found that in bulk and thin films of ZnO the UV emission can be internally reabsorbed by the crystal itself within 1μm range [17]. However, this reabsorbed UV emission can excite defect states in the material resulting in an increase of the intensity of the DBE. Thus, it is possible that part of the UV emission may contribute to the enhancement of the DBE [15]. The differences and variations in the size of the nanostructures also contribute to the emission intensities. A luminescence spectrum usually represents the optical characteristics of all the nanostructures inside the probing area, due to in-homogeneities among the nanostructures it may be considered as an average luminescence [18]. The intensity ratio of the DBE to the UV emission is very useful in exploring the origin of the deep level emission and the distribution of the recombination centers. The intensity ratio of the DBE to UV emission decreases with the penetration depth within the whole length of our ZnO NRs. From Figure 6, it is concluded that there are more deep defects at the root of ZnO NRs compared to the upper part. N. Bano *et al*. demonstrated that ZnO NRs have more structural defects at the interface of ZnO NRs and the substrates [15]. The present CL spectroscopy technique implemented here gives in depth information about the radiative defects. Using this method it is found that ZnO NRs have more radiative defects at the PFO interface. It is also proposed that some part of the UV might be reabsorbed within the ZnO NRs [3]. Confirmation of this issue requires further investigations.

**Figure 7:**
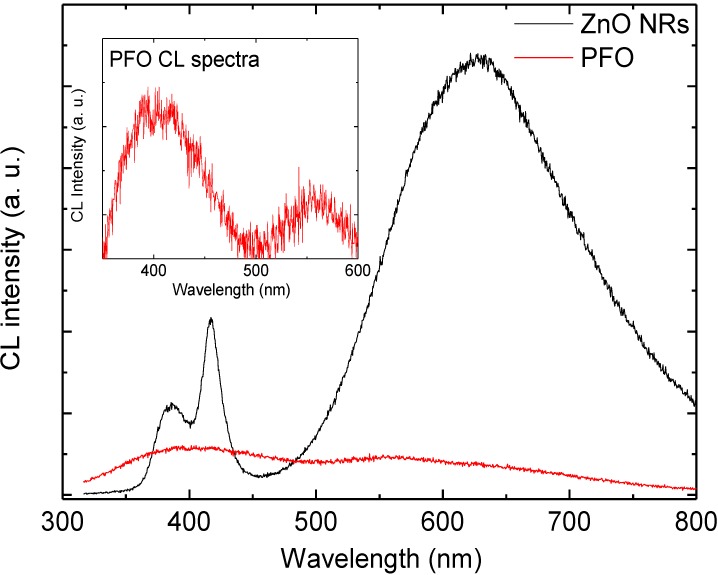
Comparison of the CL emission spectra from the hybrid LED and an area with PFO only; inset shows the magnified CL emission spectra of PFO.

We also measured a CL spectrum from an area containing no ZnO NRs, but only PFO. The CL spectra of PFO have very weak emission band centered at 400 nm and weak emission peak at 560 nm but the CL spectra of the ZnO NRs show stronger emissions at 381 nm, violet emission at 417 nm and DBE band centered at 625 nm are all present, as shown on Figure 7. The inset of Figure 7 illustrates that CL spectra of PFO have a violet-blue emission band from 350–490 nm. This confirms that the PFO have contribution to the emission in the violet-blue range. Consequently Figure 7 confirms that most of the emission observed from the ZnO NRs/PFO hybrid LED originates from the ZnO NRs, and PFO has a minute contribution of the violet-blue emission. This concludes that most of the emission in ZnO nanorods-organic hybrid white LEDs appears to originate from the ZnO NRs.

## 3. Experimental Section

To fabricate ZnO nanorods-organic hybrid white LEDs a 2 × 2 cm^2^ glass substrate, was washed in ultrasonic cleaner using isopropyl alcohol, acetone and de-ionised water sequentially. All chemicals used in the experiment were purchased from Sigma-Aldrich and were of analytical grade and used without further purification. Poly (3,4-ethylenedioxythiophene) poly (styrenesulfonate) PEDOT: PSS was then spin-coated onto the treated glass and baked at 100 °C for 5 minutes to form a uniform film. We cover small portion of the PEDOT: PSS for ohmic contact and then Polyfluorene (PFO) was spun coated on top of the PEDOT: PSS film and backed at 100 °C for 3 minutes. To grow ZnO nanorods we used the hydrothermal growth technique. In this method 0.01M zinc nitrate hexa hydrate (Zn (NO_3_).6H_2_O) was mixed with 0.01M Hexamethyl tetra-mine (HMT) (C_6_H_12_N_4_) .The sample was then placed in the solution and was heated at 90 °C for 4 hours. After growth, the samples were used to process light emitting diodes (LEDs). Prior to the ohmic contacts to the ZnO NRs, an insulating layer of spin on glass (SOG) was deposited between the NRs. To ensure that no SOG was on the top of the NRs, plasma cleaning was performed prior to the contact metal deposition. Then indium tin oxide (ITO) circular transparent contacts of 1 mm diameter and a thickness of 1000 Å were evaporated onto a group of NRs. Silver paste is used for the ohmic contact on the PEDOT: PSS. The device structure was characterized by a scanning electron microscope SEM, room temperature electroluminescence (RT-EL) and cathodoluminescence (CL). The RT-EL measurement of the ZnO nanorods-organic hybrid white LEDs was carried out using a photo multiplier detector under dc-bias conditions. The light was collected from the topside of the device. The CL spectra was measured with varying the accelerating voltage (5–20 kV), which allow probing the sample and optical emissions at different depths. However, the depth of the “generation volume” by the electron beam depends on the specific material and the electron beam energy.

## 4. Conclusions

In summary, we report white-light luminescence from ZnO nanorods-organic hybrid white light emitting diodes (LEDs) grown on glass substrate by low temperature aqueous chemical growth (ACG). The RT-EL spectra of the ZnO nanorods-organic hybrid white LEDs reveal a broad emission band from 400 to 800 nm covering the whole visible region arising from the radiative recombination in the organic PFO and ZnO NRs. To attain greater insight into the deep defect chemistry in this hybrid white LED structure, we also measured the CL spectra at room temperature (RT). The CL spectra exhibit UV emission at 381 nm and two DBE bands centered at 417 nm and 625 nm. In order to distinguish the emission components, we used a Gaussian function to simulate the EL and the CL spectra which gives clear evidence that the DBE band in both spectra have the same emission lines. Depth resolved spectroscopy was used to probe the spatial distribution of radiative defects and to investigate the self-absorption of the UV in these hybrid white LEDs structures. The CL intensities of all emission peaks have been examined as a function of the accelerating voltage. The DBE at 625 nm increases with the increase of the penetration depth due to the augmented defect concentration at the ZnO/PFO interface. The comparison of the CL spectra of the PFO and the ZnO NRs confirms that the PFO has very weak violet-blue emission band, which implies that most of the DBE originates from the ZnO NRs.
